# Pharmacophore Selection and Redesign of Non-nucleotide Inhibitors of Anthrax Edema Factor

**DOI:** 10.3390/toxins4111288

**Published:** 2012-11-08

**Authors:** Catherine H. Schein, Deliang Chen, Lili Ma, John J. Kanalas, Jian Gao, Maria Estrella Jimenez, Laurie E. Sower, Mary A. Walter, Scott R. Gilbertson, Johnny W. Peterson

**Affiliations:** 1 Sealy Center for Structural Biology and Molecular Biophysics, Department of Biochemistry and Molecular Biology, University of Texas Medical Branch, Galveston, TX 77555, USA; Email: deliang2211!@hotmail.com; 2 Sealy Center for Vaccine Development, Center for Biodefense and Emerging Infections, University of Texas Medical Branch, Galveston, TX 77555, USA; Email: jpeterso@utmb.edu; 3 Department of Microbiology and Immunology, University of Texas Medical Branch, Galveston, TX 77555, USA; 4 Member, Institute for Translational Studies, and Faculty, Institute for Human Infection and Immunity, University of Texas Medical Branch, Galveston, TX 77555, USA; 5 Department of Chemistry, University of Houston, Houston, TX 77004, USA; Email: mal1@nku.edu (L.M.); mestrell@hotmail.com (M.E.J.); srgilbe2@central.uh.edu (S.R.G.); 6 Mission Pharmacal Company, San Antonio, TX 78230, USA; Email: jkanalas@satx.rr.com (J.J.K.); jian.gao@missionpharmacal.com (J.G.); mary.walter@missionpharmacal.com (M.A.W.); 7 Chrysalis Biotherapeutics, Galveston, TX 77555, USA; Email: lauries806@gmail.com

**Keywords:** computational design, library screening, fluorenone, adenylyl cyclase toxin

## Abstract

Antibiotic treatment may fail to protect individuals, if not started early enough, after infection with *Bacillus anthracis*, due to the continuing activity of toxins that the bacterium produces. Stable and easily stored inhibitors of the edema factor toxin (EF), an adenylyl cyclase, could save lives in the event of an outbreak, due to natural causes or a bioweapon attack. The toxin’s basic activity is to convert ATP to cAMP, and it is thus in principle a simple phosphatase, which means that many mammalian enzymes, including intracellular adenylcyclases, may have a similar activity. While nucleotide based inhibitors, similar to its natural substrate, ATP, were identified early, these compounds had low activity and specificity for EF. We used a combined structural and computational approach to choose small organic molecules in large, web-based compound libraries that would, based on docking scores, bind to residues within the substrate binding pocket of EF. A family of fluorenone-based inhibitors was identified that inhibited the release of cAMP from cells treated with EF. The lead inhibitor was also shown to inhibit the diarrhea caused by enterotoxigenic *E. coli* (ETEC) in a murine model, perhaps by serving as a quorum sensor. These inhibitors are now being tested for their ability to inhibit Anthrax infection in animal models and may have use against other pathogens that produce toxins similar to EF, such as *Bordetella pertussis* or *Vibrio cholera*.

## 1. Introduction

The morbidity and mortality of many pathogenic bacteria is due to the production of toxins, important virulence factors with many different basic enzymatic activities. Even after treatment of systemic infection with *Bacillus anthracis*,the causative agent of anthrax, patients continued to suffer the effects of the toxins. For example, several people died in hospitals after the bioweapon attacks of 2001, despite administration of antibiotics [1]. These unfortunate events highlighted the potential use of *B. anthracis*, an NIAID category A pathogen, as a bioweapon, and lead to greater interest in developing inhibitors of the toxins it produced, as well as several other toxins from NIAID category B Biodefense target bacteria. 

The complex produced by *B. anthracis* consists of a virulent mixture of two toxins, with different enzymatic activities, both of which have a similar *N*-terminal domain that allows them to bind to a large protein, Protective Antigen (PA) for receptor binding and cell entry [2,3]. Alone, neither factor can enter cells [4], so many groups have focused on inhibiting the pore forming ability or toxin binding surface of PA [5,6,7]. While both enzymatic factors have several domains, the active moiety of Lethal Factor (LF) is Zn^2+^ metalloprotease. Edema Factor (EF) is an adenylyl cyclase that catalyzes the conversion of ATP to 3',5'-cyclic adenosine monophosphate (cAMP). Both toxins can be reconstituted *in vitro*, by combining PA with LF or EF, to form lethal toxin (LT) or edema toxin (ET) [8]. This is the basis of cell-based assays for toxin inhibitors that are described in more detail below.

Specific inhibitors of the metalloprotease site of LF have been selected by other groups [9,10,11,12,13]. Thanks to initial generous funding from the DOD, NIH, and Mission Pharmacal, our group was able to focus on identifying non-nucleotide inhibitors of EF. We chose to do a direct design, based on analysis of the structure of the substrate binding site of the EF protein, rather than one starting from modifying nucleotides related to the substrate itself [14]. These results, and the initial success of the inhibitors in *in vitro* cell assays and *in vivo* assays in inhibiting diarrhea in mice, are described here. Below we summarize the key steps in identifying EF inhibitors, and the biological experiments that document their usefulness for treating bacterial intoxication. The process is broken down into the basic stages of the discovery process below.

## 2. Pathway to Discovering a Family of Inhibitors of EF

### 2.1. Studying the Active Site of EF

**Figure 1:**
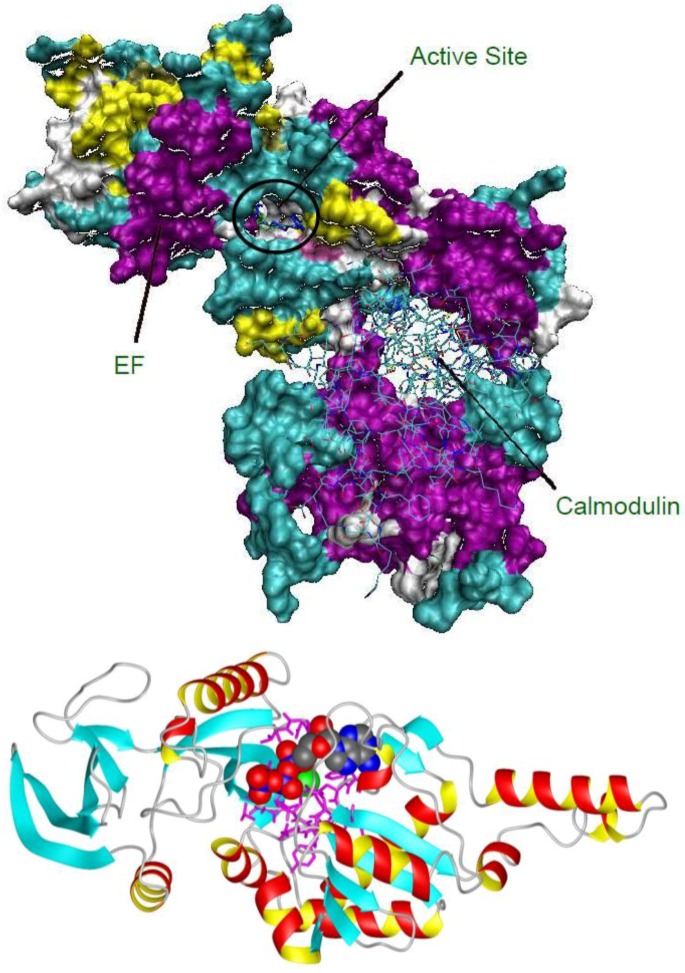
(**Top**) The overall structure of anthrax EF (plus calmodulin [17]) indicating the small area targeted by the inhibitors in this study; (**Bottom**) detail of the adenylyl cyclase domain of 1K90.pdb, with the Yb ion (green), and the inhibitor included in the co-crystal structure (3'dATP, colored according to atom type) shown as space filling. The magenta lines indicate residues of EF that surround the active (substrate binding) site.

Analysis of crystal structures of EF with various substrate analogues was the first step in our design process. EF can be allosterically activated by the presence of other proteins, such as calmodulin, which is a Ca^2+^ ion sensor present in host cells. Inhibitors targeting sites for such allosteric activators have recently been identified [15]. Our studies focused on the active site (circled in the structure of EF bound to calmodulin, shown in Figure 1Top). Comparison of the active site conformation in various crystal structures in the Protein database (PDB) (which differed in the number and types of bound metal ions and substrates [16]) revealed important information about how the active site of the toxin differed from the mammalian adenyl cyclase enzymes. These crystal structures, with or without the bound metal ions, were used for docking potential inhibitors identified by our fragment based pharmacophore.

**Figure 2:**
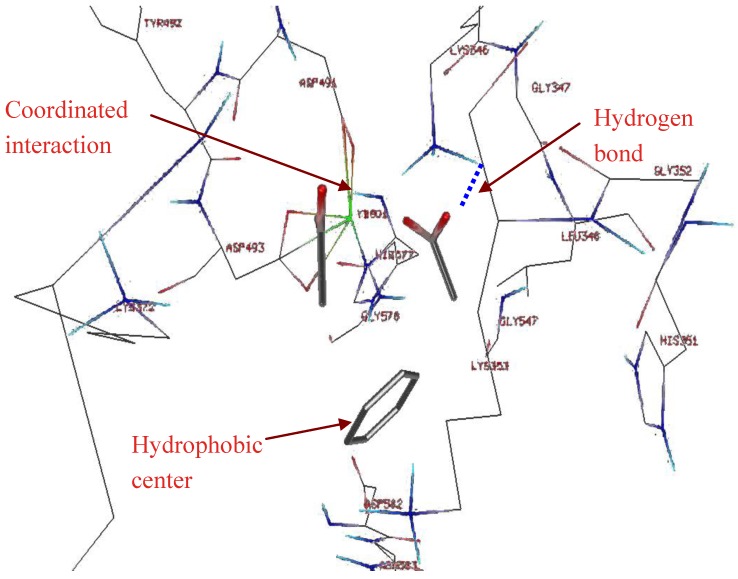
Design of a fragment based pharmacophore using the HINT (Hydropathic INTeractions) program, the lowest energy binding sites of a benzene ring, and two carboxyls and the distances between the three fragments are the basis of a 3D-pharmacophore, suitable for compound library screening with the Unity program. Note that HINT was used again to determine the optimal binding site of larger fragments, as described in Figure 4.

### 2.2. Compound Library Screening with a Fragment Based, 3D-Pharmacophore

**Figure 3:**
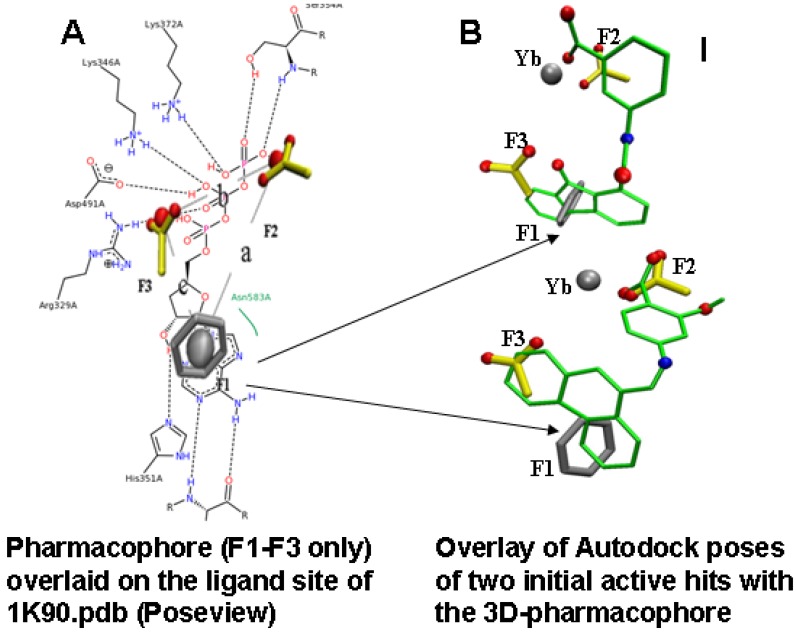
Overview of the fragment based pharmacophore design. (**A**) Overlay of the initial 3D-pharmacophore designed based on the HINT selected fragments (Figure 2; F1: phenyl ring; F2, F3 carboxyl groups, with distance constraints a, b, c) on a 2D image of the ligand binding site (for 3'dATP) of 1K90 (Poseview [21])); (**B**) Shows the overlay of the pharmacophore with docking poses (to the 1K90 structure, with the substrate removed) for two of the active compounds identified in the first bioscreening (3-[(9-oxo-9*H*-fluorene-1-carbonyl)-amino]-benzoic acid, top and 4-[(anthracen-9-ylmethylene)-amino]-2-hydroxy-benzoic acid, bottom). These dockings indicate that the orientation was consistent with the fragment positions in the active site. Compound 1(top), a substituted fluorenone, was our lead for subsequent redesign.

A fragment library was built that contained small molecules with at most one rotatable bond. The HINT program was used to select those fragments that bound to areas in the active site of EF. The Hydropathic INTeractions, or HINT, program [18,19,20] uses experimental solvent partitioning data as a basis for calculating free energy scores of binding. Interaction energy calculations used to score fragment binding included terms for hydrophobic, ionic, and hydrogen bond interactions (Figure 2 and Figure 3). Initially, a smaller library, from the NCI, was screened with the pharmacophore and 8 compounds selected from this list that had particularly good scores with the FlexX docking program. Then these compounds were used to identify larger fragments that were used to screen the ZINC library for compounds.

**Figure 4:**
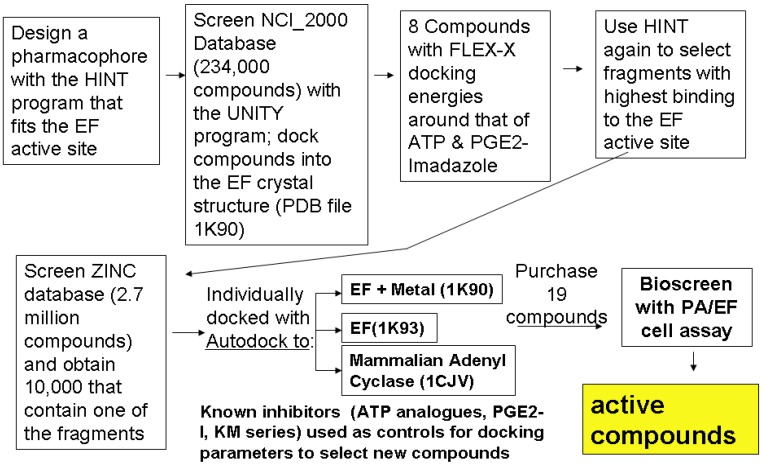
Overall scheme for selecting inhibitors of EF using compound library screening and docking with Flex-X and Autodock. Note the HINT program was used at two levels. In the first, simple fragments were used to design a 3-D pharmacophore, and select a small group of compounds with Flex-X. These compounds were then analyzed to find larger common fragments that were again used to refine the pharmacophore. Several versions of these pharmacophores were then used to screen ZINC [22] to obtain 10,000 compounds that were ranked by docking (to several different crystal structures from the PDB) with Autodock3. Purchased compounds were tested as described in the next section.

### 2.3. Compound Selection Using Molecular Docking

Molecular docking played an important role in our identification of a novel inhibitor of EF, compound 1 [23] (Figure 3B top), when used in combination with pharmacophore-based compound selection, and experimental screening with a cell-based bioassay [16,24]. Autodock was used for this work, after we compared several different docking programs for how well they were able to reproduce the position within the active site of analogues of ATP compared to that of the crystal structure. This work revealed that that the protonation state of inhibitors had a pronounced effect on docking, consistent with work by others [25]. From about 5000 compounds chosen in our initial screening of the NCI and ZINC libraries, about 20 purchasable compounds were assayed as described below. Of these, three compounds were active at concentrations below 10 µM in the cell assay. One of these, (compound 1) proved to be an effective lead compound, as its activity was reproducible; it had reasonable (but not ideal) aqueous solubility, and showed no toxicity in our initial tests. 

## 3. Bioassay for Activities, Toxicities

### 3.1. Experimental Assay for Inhibition of Toxin Induced Release of cAMP

All compounds and synthesized derivatives were dissolved in DMSO and then diluted at least 100 fold into cell culture medium before assay. Their ability to reduce total extracellular cAMP production in mammalian cells treated with EF/PA was determined. Assays were done in triplicate and samples with IC_50_ values < 20 μM were assayed at least twice on different days, and re-assayed in 2-fold dilution steps to reduce errors at lower doses. 

**Figure 5:**
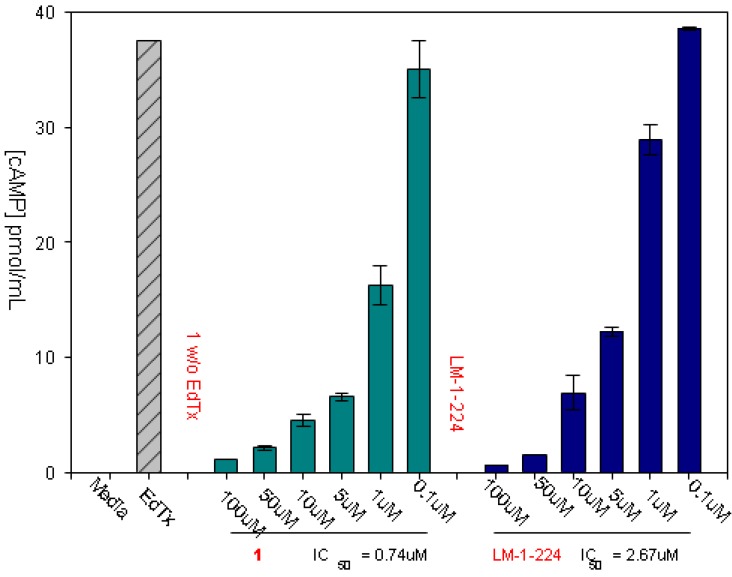
Assaying compounds for inhibition of EF induced extracellular cAMP secretion, used RAW 264.7 cells treated with edema toxin (30 nM PA and 70 nM EF). Cells were incubated with the indicated concentrations of our lead inhibitor (Figure 3B, top) and a derivative, LM-1-224 (2-Hydroxy-5-(9-oxo-9H-fluorene-1-carboxamido)benzoic acid). The first bar in each series shows that negligible cAMP is induced when cells are treated with the compounds alone, without EF addition. Cells were incubated for 4 h before assay. cAMP was measured with the Assay designs, Inc direct cAMP kit.

Our standard assay for the activity of the selected inhibitors was inhibition of cAMP released to the medium of cells treated with edema toxin (EF + PA). An example of the assay, for two of our inhibitors, is shown in Figure 5.

Our reliance on this assay was for several reasons. First, experience has shown that direct inhibitors of enzyme activity can fail at the level of the whole cell, due to inability to enter the cell, or problems with toxicity that may be direct effects or due to off target effects on other cellular enzymes. Further, the cell assay was exquisitely sensitive, able to measure the effects of very small amounts of EF (administered with PA). This assay gives more variability than using isolated enzymes, but it is biologically more relevant, as the concentrations needed to inhibit diarrhea and intestinal damage in the ETEC murine model (7.5–15 μg/mouse) were similar to those indicated by the cell culture IC_50_ values for compound 1 [26], as discussed below. 

### 3.2. Treating Enterotoxigenic *E. coli* (ETEC) Infections in a Murine Model

Due to the cost of testing the inhibitors against *B. anthracis* infection, assays for which must be done in BSL-3 conditions, a BSL-2 experiment was conducted to determine whether our inhibitors could prevent intestinal edema and diarrhea during entertoxigenic *E. coli* (ETEC) infection in mice. This murine model of bacterial infection was used as ETEC produce an adenylyl cyclase toxin that has a high degree of identity to EF, known as heat-labile enterotoxin (LT) [27]. ETEC is a leading cause of traveler’s diarrhea [28,29]. Periodic outbreaks occur in the developing world [30] and with increasing frequency in the US [31,32]. A murine model was developed to test the effect of our inhibitors on the progress of the infection, and particularly development of diarrhea, using a gavage method to infect the animals, with the inhibitor supplied intraperitoneally both before and after the inoculation of the mice. In this minimally invasive model, the flow in the intestine was not interrupted, and it thus approximated that of a natural infection. Our lead compound significantly decreased intestinal colonization of ETEC in this model. However, the toxin inhibitor did nothing to inhibit the growth of several different pathogenic bacteria in flask culture [26]. This example illustrates the need for testing toxin inhibitors in animal models, as in this case the cAMP secretion induced by the toxin might have served to inhibit cell attachment by the bacteria [27] or as a quorum sensor that stimulates bacterial replication [33]. This sort of activity would not be clear from its effects on bacteria growing rapidly in normal culture medium, where the toxin would not be required as a growth stimulator [34].

### 3.3. Additional Assays for Toxicity, and Estimation of Solubility

Compound 1 inhibited cAMP production induced by PA and EF (IC_50_ 2–9 μM in cells) [35] and reduced bacterial colonization and diarrhea caused by ETEC in mice with an oral gavage dose of 15 μg/mouse. While there was no obvious toxic effect in the cell cultures or mouse studies, and the Ames II™ Mutagenicity Assay determined the compound 1 to be non-mutagenic in all conditions tested, the compound gave a positive response for genotoxicity in a Green Screen HC [GADD45α (growth arrest and DNA damage gene)-Green Fluorescent Protein (GFP)] assay test on mammalian cells at 10–20× its active dose (62.5 µg/mL *vs.* active dose in mice of about 3 µg/mL, assuming 5 mL serum/mouse).

## 4. Redesign of the Inhibitors for Enhanced Solubility and Reduced Potential Toxicity

Our previously identified lead compound 1 was active in bioassays in the low micromolar range, and the effective dose to reduce diarrhea in mice (from ETEC) was also low, on the order of 7.5–15 μg when given intraperitoneally [26]. While there was no obvious toxicity to the mice, there was some indication of cytotoxic potential in the “Green Screen” assay at levels 10–20× the active concentration. Our initial results indicated that the fluorenone ring contributed to the inhibitory activity [36], and thus, most of the derivatives synthesized targeted the benzoic acid side chain, which had some markers of toxicity [37]. We were able to identify several derivatives with better predicted pharmaceutical properties, and equivalent or better activity in the bioassay for cAMP production induced by treatment of mammalian cells with ET. Derivatives of compound 1 were designed to add substituents at positions that had contact with residues important in the active site, according to docked conformations. The designed compounds were then redocked with AutoDock 3.0 [38,39]. Our previous studies [16] indicated that the docking of 3'-dATP to the crystal structure of EF (PDB structure: 1K90 [17]) had low RMSD (root-mean-square deviations) between the predicted structure and the crystal structure, and thus, AutoDock 3.0 was reliable enough to predict the binding mode of the ligands to EF. 

Of the compounds selected for synthesis and assay, those with hydrogen bond acceptors and hydrogen bond donors added to the benzoic acid improved the predicted clogP. Perhaps the biggest surprise from the derivatives was that we could substitute the benzoic acid with 3-carbamimidoylphenyl, without losing activity. We had initially assumed that the carboxyl group of the benzoic acid was essential, as methoxy analogues did not have as high an inhibitory activity as their carboxyl-counterparts. Further, all of our dockings of compound 1 indicated that the carboxyl (as shown in Figure 3B, where the benzoic acid carboxyl overlays F2) interacted with the metal ion and/or positively charged residues such as Arg329, Lys346, Lys353, and Lys372 in the active site of EF. We thus considered that this group should bind in a similar position as the phosphate oxygens of 3'-dATP, and that it would be essential for activity. Further docking analysis of the compound containing the carbamimidoylphenyl derivative indicated that this side chain could target another, negatively charged area of the active site that we had not included in the initial pharmacophore, specifically the backbone oxygen atoms from the residues of Lys346, Gly347, Val350 and carboxyl group of Asp491 [37]. The carbamimidoylphenyl derivative, as it has a lower clogP than compound 1, and no markers of toxicity, has now been included for future animal testing.

## 5. Conclusions

Although early treatment with antibiotics can greatly reduce the effects of bacterial infections, inhibitors of the toxins could play a therapeutic role in later stage infections, in preventing toxin induced diarrhea or even death. Here we showed how a group of novel inhibitors of EF, based on an initial hit, a fluorenone compound 1 (Figure 3B top), was identified using a combination of pharmacophore-based compound selection, molecular docking, and experimental screening with a cell-based bioassay. This is a work in progress, and many assays and GLP studies remain to be done to definitively document the usefulness of the compounds as human therapeutics. Further, while our results show that the fluorenone based inhibitors can be useful on their own, an ideal therapy could be to combine them with LF inhibitors to treat late stage anthrax infections, where antibiotics might be unable to prevent death. These EF inhibitors could also be useful against other bacterial pathogens such as ETEC, *Bordetella pertussis*, the causative agent of whooping cough, and *Vibrio cholera*, all of which produce toxins similar to EF. 
